# Molecular Variability and Host Distribution of ‘*Candidatus* Phytoplasma solani’ Strains from Different Geographic Origins

**DOI:** 10.3390/microorganisms9122530

**Published:** 2021-12-07

**Authors:** Nicoletta Contaldo, Jelena Stepanović, Francesco Pacini, Assunta Bertaccini, Bojan Duduk

**Affiliations:** 1 Department of Agricultural and Food Sciences, Alma Mater Studiorum-University of Bologna, 40127 Bologna, Italy; francesco.pacini2@unibo.it (F.P.); assunta.bertaccini@unibo.it (A.B.); 2 Institute of Pesticides and Environmental Protection, 11080 Belgrade, Serbia; Jelenamitrovic@pesting.org.rs (J.S.); bojan.duduk@pesting.org.rs (B.D.)

**Keywords:** phytoplasma disease, molecular characterization, genetic diversity, ecology, epidemiology

## Abstract

The knowledge of phytoplasma genetic variability is a tool to study their epidemiology and to implement an effective monitoring and management of their associated diseases. ‘*Candidatus* Phytoplasma solani’ is associated with “bois noir” disease in grapevines, and yellowing and decline symptoms in many plant species, causing serious damages during the epidemic outbreaks. The epidemiology of the diseases associated with this phytoplasma is complex and related to numerous factors, such as interactions of the host plant and insect vectors and spreading through infected plant propagation material. The genetic variability of ‘*Ca*. P. solani’ strains in different host species and in different geographic areas during the last two decades was studied by RFLP analyses coupled with sequencing on *vmp1*, *stamp,* and *tuf* genes. A total of 119 strains were examined, 25 molecular variants were identified, and the variability of the studied genes was linked to both geographic distribution and year of infection. The crucial question in ‘*Ca*. P. solani’ epidemiology is to trace back the epidemic cycle of the infections. This study presents some relevant features about differential strain distribution useful for disease monitoring and forecasting, illustrating and comparing the phytoplasma molecular variants identified in various regions, host species, and time periods.

## 1. Introduction

“Bois noir” (BN), the most widespread grapevine yellows disease, represents a worldwide threat to viticulture. It is associated with the presence of ‘*Candidatus* Phytoplasma solani’ [1], an obligate cell-wall-lacking bacterium that belongs to the class *Mollicutes* and is transmitted by polyphagous phloem-feeding insects [2,3]. It is enclosed in the 16SrXII-A ribosomal subgroup and associated also with the “stolbur” disease in vegetable crop species, mostly belonging to the *Solanaceae* (tomato, potato, and pepper) and *Apiaceae* (carrot, celery, and parsley) families [4,5]. Due to its complex ecology comprising diverse insect vectors and a broad range of host plant species, it is difficult to design effective strategies for the management of both “bois noir” and “stolbur” diseases. Insect vectors represent one of the critical points in the spread of this phytoplasma. The polyphagous cixiid *Hyalesthes obsoletus* Signoret transmitting the phytoplasma is ubiquitous in Europe to a wide range of wild and cultivated plants [6,7,8,9,10], which in return represent a reservoir of the pathogen in and outside the cultivated fields. *Reptalus panzeri* and *R*. *quinquecostatus* have been reported as vectors of BN in Serbian and French vineyards, respectively [11,12], while *Anaceratagallia ribauti* was reported as vector of “stolbur” to broad bean plants [13]. Other studies described the ability of *R. panzeri* collected in maize fields with a reddening disease to transmit ‘*Ca*. P. solani’ to grapevine plants [11]. Recent transmission trials conducted with insects collected in a Northern Italy vineyard showed that at least eight insect species (*Aphrodes makarovi*, *Dicranotropis hamata*, *Dictyophara europaea*, *Euscelis incisus*, *Euscelidius variegatus*, *Laodelphax striatellus*, *Philaenus spumarius*, and *Psammotettix alienus/confinis*) are vectoring BN [14], therefore confirming the complex epidemiology of ‘*Ca*. P. solani’-associated diseases [1]. Moreover, a large genetic diversity was described for this phytoplasma after grapevine-infecting strain molecular characterization on multiple genes (i.e., *tuf*, *secY*, *vmp1*, and *stamp*), highlighting the presence of many genetic lineages or variants [15,16,17], and of a positive selective pressure determining the strain population complexity in different vineyard agroecosystems [18]. One of the first genes used for epidemiological studies is the housekeeping gene *tuf* (elongation factor Tu), of which four variants and several subvariants (*tuf* types) were described in Europe [19,20,21,22,23]. ‘*Ca*. *P. solani*’ *tuf* type b1 was mainly identified in *Hyalesthes obsoletus* and *Convolvulus arvensis*, *Vitex agnus-castus* and *Crepis foetida*, and *Reptalus panzeri* [20,21,22]. *Tuf* types a and b2, harbored by *Urtica dioica,* are reported to be only transmitted by *H. obsoletus*. A *tuf* type c was erratically detected in hedge bindweed (*Calystegia sepium*) in a restricted area of Germany [19,24]; a *tuf* type b3 variant was reported in vineyards in the Republic of Azerbaijan [25]; and a *tuf* type d was very recently described in Serbia, in a few crop species (sugar beet, parsnip, and parsley) [23].

Molecular epidemiological studies focused on the distribution of BN and “stolbur” strains in their hosts (plants and insects) increased the knowledge about their transmission in vineyard agroecosystems and natural environments. Recently, the use of several molecular markers suggest a possibility to differentiate BN strains for their differential virulence in grapevine plants [17,26]. However, in most cases molecular markers are mainly used in combination to resolve epidemic cycles at a regional level [11,20,21,24,27,28,29,30].

Multilocus sequence typing (MLST), based on molecular characterization of more variable genes such as *vmp1* and *stamp*, evidenced a large variability among ‘*Ca*. P. solani’ strains within the *tuf* types [17,31]. Molecular approaches, using vmp1- and stamp-based molecular markers, allowed the increase of the knowledge of these phytoplasma population structures and dynamics [17,32] and their transmission routes throughout vineyards and their surroundings [21,33].

In the present study, a characterization of ‘*Ca*. P. solani’ strains collected in the last two decades in different European regions and from different host species was carried out by RFLP analyses and sequencing of *tuf*, *stamp,* and *vmp1* genes to verify possible correlation between the variants and the disease outbreaks towards designing focused monitoring and control strategies.

## 2. Materials and Methods

### 2.1. Sources of Nucleic Acid

In this study, 119 ‘*Ca.* P. solani’ strains identified on the 16S ribosomal gene (data not shown) collected during 20 years from nine European countries (Serbia-46, Italy-47, Hungary-10, Portugal-7, Bulgaria-4, France-2, Montenegro-1, Spain-1, and Slovenia-1) from 15 naturally infected plant species (grapevine-54, tomato-13, periwinkle-9, corn-6, parsley-5, parsnip-4, potato-4, bindweed-4, celery-3, pepper-3, tobacco-3, valerian-3, carrot-2, *Parthenocissus quinquefolia*-2, and *P. tricuspidata*-1) and the insect vector *H. obsoletus* (3) collected in or near vineyards were employed. Total nucleic acids were extracted using a phenol/chloroform (C/P) [34] or a CTAB [35] protocol. The extracted DNAs were diluted to 20 ng/μL (C/P) and 1 to 100 (CTAB) with sterile deionized water (SDW) for direct PCR assays, and subsequently the amplicons were diluted 1:30 for nested PCRs; 1 μL was used as a template for the PCR and nested PCR procedures. The phytoplasma strains MOL and STOF (from France), STOL (from Serbia), ASLO (from Slovenia), and P-TV (from Italy), all from the periwinkle (*Catharanthus roseus* (L.) G. Don) collection maintained at the University of Bologna [36], were used as positive controls.

### 2.2. Amplification of ‘Ca. P. solani’ Strains

The genes *tuf*, *stamp,* and *vmp1* were studied for molecular differentiation of the ‘*Ca*. P. solani’ strains used. All the PCRs were performed in a final volume of 25 μL containing 12.5 μL of PCR Master Mix (2X) (Fermentas, Lithuania, 0.05 U/μL *Taq* DNA polymerase, reaction buffer, 4 mM MgCl_2_, and 0.4 mM of each dNTP), 10.5 μL of SDW, 0.5 μL of each primer at 20 pmol/μL (final concentration 0.4 μM), and 1 μL DNA template (20 ng). Positive controls were used in all PCR amplifications. Samples containing SDW as a template were used as negative control in both the PCR and nested PCR assays.

The *tuf* gene was amplified using the primer pairs fTuf1/rTuf1 and fTufAy/rTufAy in nested PCR [37]. The *stamp* gene was amplified with primers StampF and StampR0 and the nested primers StampF1 and StampR1 following described reaction conditions [15]. The *vmp1* gene was amplified with H10F1/R1 [38], followed by nested PCR with the TYPH10F/R primer pair [39]. A 6 μL aliquot of PCR products was separated by electrophoresis through 1% agarose gel, stained with ethidium bromide, and visualized with UV transilluminator with a 1 kb DNA ladder (Bioline, England) as marker.

### 2.3. Restriction Fragment Length Polymorphism (RFLP) Analyses

RFLP analyses of the *tuf*, *stamp,* and *vmp1* gene amplicons were performed using *Hpa*II, *Tru1*I, and *Rsa*I restriction enzymes, respectively. All the enzymes were from Thermo Fisher, Lithuania, and were used according to the manufacturer’s instructions. Obtained restriction products were separated by electrophoresis in 6.7% or 8% polyacrylamide gel, stained, and visualized as described above, using the ΦX174/*Hae*III DNA ladder (Fermentas, Lithuania) as a marker. To verify the accuracy in the determination and recognition of the different RFLP patterns obtained in the PCR⁄RFLP analysis, the pDRAW32 software (AcaClone software, http://www.acaclone.com (accessed on 15 September 2021)) was used for virtual digestion of the *vmp1* and *stamp* sequenced amplicons with the *Tru1*I and *Rsa*I endonuclease, respectively. *Tuf* amplicons showed RFLP profiles less variable, therefore the attribution to *tuf* variants was made based on RFLP and sequences similarity.

### 2.4. Sequencing and Phylogenetic Analyses

Direct sequencing of 88 amplicons from the different genes (9 *tuf*, 47 *stamp,* and 32 *vmp1*) selected considering the RFLP profiles, the host species, and the quality of the amplicons bands in the agarose gel, was performed by Macrogen Inc. (Netherlands) on both strands, using the same primers employed for the amplification. Raw sequences were assembled and edited using Pregap4 and Gap4 software from the Staden package [40], and the representative ones were deposited in GenBank database. Nucleotide sequences were compiled in FASTA format, and multiple alignments were performed with ClustalW [41]. The *vmp1* gene sequences were trimmed to approximately 1,300 nt and the *stamp* gene sequences to approximately 500 nt, and phylogenetic analyses were carried out with MEGA X [42] using the neighbor-joining method [43], with 1000 bootstrap replicates to estimate the solidity of the analysis. Phylogenetic trees were constructed based on nucleotide sequences of *vmp1* and *stamp* genes produced in this work, strain’s sequences from previous studies [17,26,44,45] and retrieved from the NCBI GenBank (Table 1). *Stamp* gene nucleotide sequences were analyzed by sequence identity matrix to calculate their genetic diversity and aligned with 70 sequences of previously defined *stamp* sequence variants [26,44,45]. A nucleotide sequence identity of 100% was employed for the sequence variant attribution.

## 3. Results

The 119 ‘*Ca*. P. solani’ strains tested provided amplification on the *stamp* and *vmp1* genes in 111 samples, while the *tuf* gene was positive in 108 samples. Readable RFLP profiles for all three genes were obtained for 94 strains (Table 2), while for 25 samples, one or two genes did not give amplification or the RFLP profile was inconclusive. Twenty samples gave amplification on two genes, while five grapevine samples were amplified only on one gene, indicating a different rate of amplification according with the gene employed.

RFLP and sequencing analysis on the *tuf* gene showed the prevalence of the *tuf* type b1 profile [19,20] identified in samples from Serbia, Italy, Spain, Portugal, Montenegro, and Bulgaria, while *tuf* type b2 was only found in two grapevine samples from Hungary. Additionally, 15 grapevine samples from Italy, mainly collected in 2010 and 2020 and one sample collected in 2019, showed a *tuf* type a profile, which was also identified in *Parthenocissus* spp. from Italy in 2005, 2018, and 2020 (Table 2).

**Figure 1:**
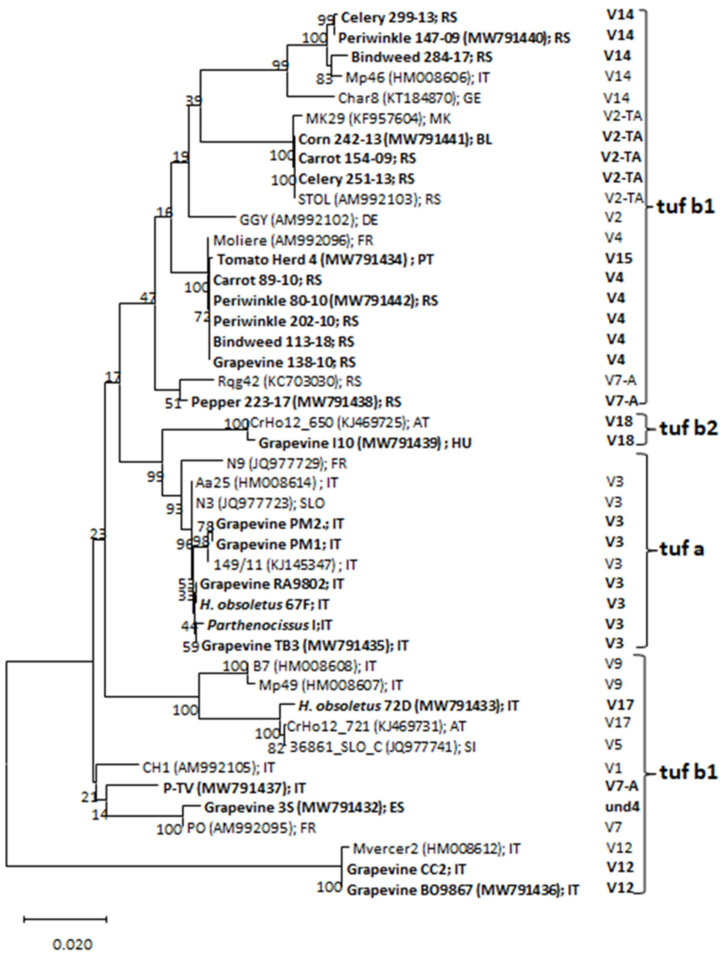
Unrooted phylogenetic tree inferred from the ‘*Ca.* P. solani’ strain nucleotide sequences of the *vmp1* gene. The phylogenetic analysis was carried out using the neighbor-joining method and bootstrap-replicated 1000 times. The phytoplasma strain acronyms are given in the tree. The GenBank accession number of each sequence is given in parentheses; gene sequences obtained in the present study are indicated in bold. *Rsa*I-V *vmp1* gene profiles are reported next to the tree. Clusters according to *tuf* types are also shown on the right. AT, Austria; BL, Bulgaria; DE, Germany; ES, Spain; FR, France; GE, Georgia; HU, Hungary; IT, Italy; MK, Macedonia; PT, Portugal; RS, Serbia; SLO, Slovenia.

**Figure 2:**
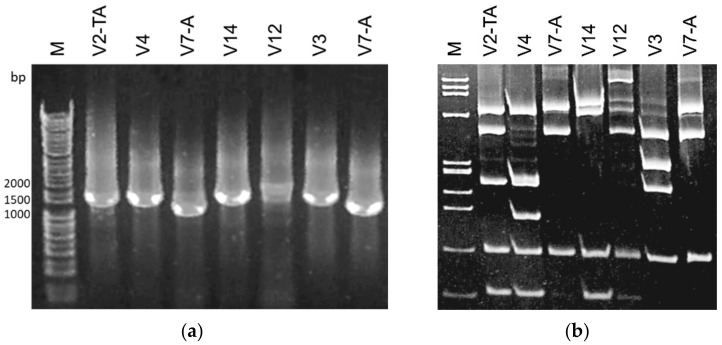
(**a**) Agarose gel (1%) showing representative amplicon sizes of the *vmp1* gene obtained with the nested primer pair TYPH10F/R. In lanes V2-TA, V4, V14, and V3: amplicons of about 1450 bp; in lanes V7-A: amplicons of about 1200 bp; in lane V12: an amplicon of about 1700 bp. M: Ladder, 1 kb DNA; (**b**) Polyacrylamide gel (6.7%) showing representative *Rsa*I RFLP patterns of digested *vmp1* gene amplicons obtained with the primer pair TYPH10F/R. M: Ladder, ΦX174 *Hae*III digested with fragment sizes in base pairs from top to bottom of 1353, 1078, 872, 603, 310, 281, 271, 234, 194, 118, and 72.

A phylogenetic tree was constructed with 26 *vmp1* gene sequences representing the different RFLP profiles observed, and 18 strains retrieved from NCBI GenBank database representing the *vmp1* gene profiles according to the literature [11,28]. The sequences generally clustered according to the RFLP profiles (Figure 1). Only the two samples (strain P-TV from Italy and pepper 223-17 from Serbia) that exhibited a 1,200 bp fragment after nested TYPH10F/R PCR on *vmp1* gene showed an identical *Rsa*I restriction profile (Figure 2); while in the phylogenetic tree, they appeared to cluster separately (Figure 1). These two strains were differentiated by *Alu*I virtual digestion (data not shown) and resulted in the V7-A and V7 profiles, respectively [11]. The enzymatic digestion with *Rsa*I on *vmp1* gene amplicons allowed the identification of 14 RFLP profiles (Table 2), according to 23 V-types reported in previous studies [11,28,31]. Furthermore, three *vmp1* gene amplicon sizes were obtained, approximately 1,700, 1,450, and 1,200 bp long. The largest polymorphism was found in the 1,450 bp amplicons, for which 10 RFLP profiles were differentiated (V2-TA, V3, V4, V11, V14, V15, V18, V17, und1, und2, and und3); on the other hand, only the profiles V7 and V7-A from the shortest amplicon and V11 and V12 from the longest were detected (Figure 2).

The RFLP analysis conducted on *stamp* gene amplicons revealed the presence of five profiles (Table 2). A phylogenetic tree was constructed using representative nucleotide sequences of the *stamp* gene obtained in this study and 70 stamp sequences retrieved from previous studies [19,27,44,46]. The phylogenetic analysis showed the presence of 11 *stamp* variants (St1-3, St2-11, St3-2, St4-3, St5-8, St8-4, St9-1, St10-2, St11-2, St18-2, and St19-6) determined by comparison with the available *stamp* gene dataset [44,46]. Two variants identified in tomato from Portugal and grapevine from Spain were found for the first time in the present study and were deposited at NCBI GenBank with the accession numbers MW759855 (tomato P3) and MW759856 (grapevine 3S) (Table 3). 

**Figure 3:**
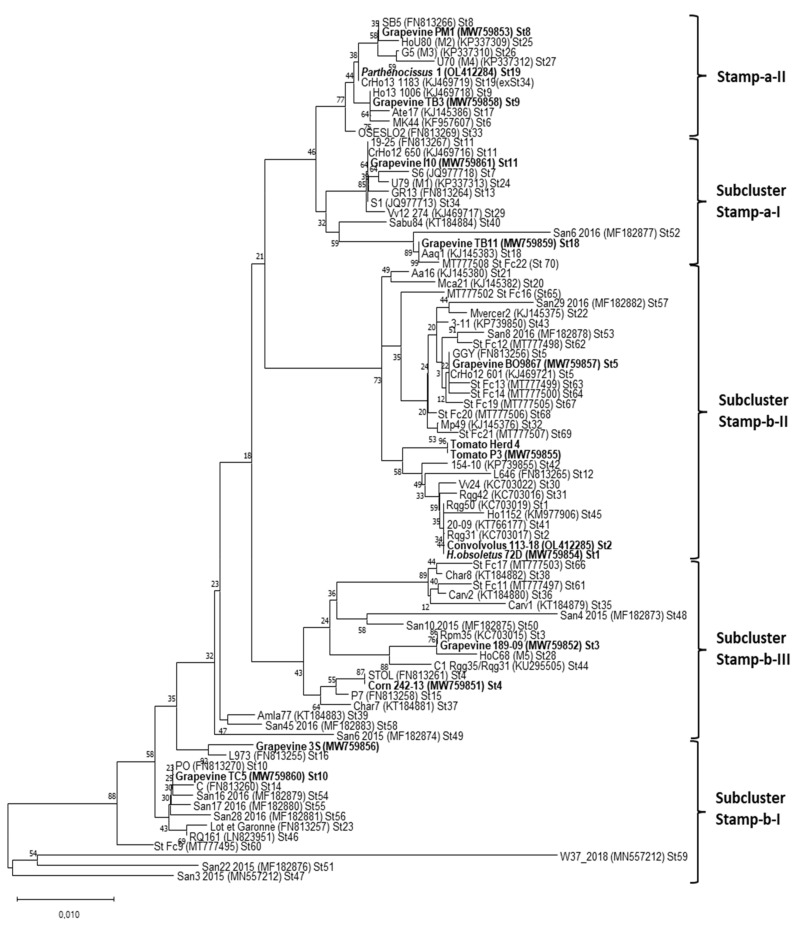
Unrooted phylogenetic tree inferred from *stamp* gene nucleotide sequences of ‘*Ca*. P. solani’ strain representative of *stamp* sequence variants previously described [17,26,41,42] and identified in this work (Table 3). Phylogenetic analysis was carried out using the neighbor-joining method and bootstrap-replicated 1000 times. Phytoplasma strains included in the phylogenetic analysis are given in the tree image. The GenBank accession number of each sequence is given in parentheses; gene sequences obtained in the present study are indicated in bold. Clusters are shown on the right.

**Figure 4:**
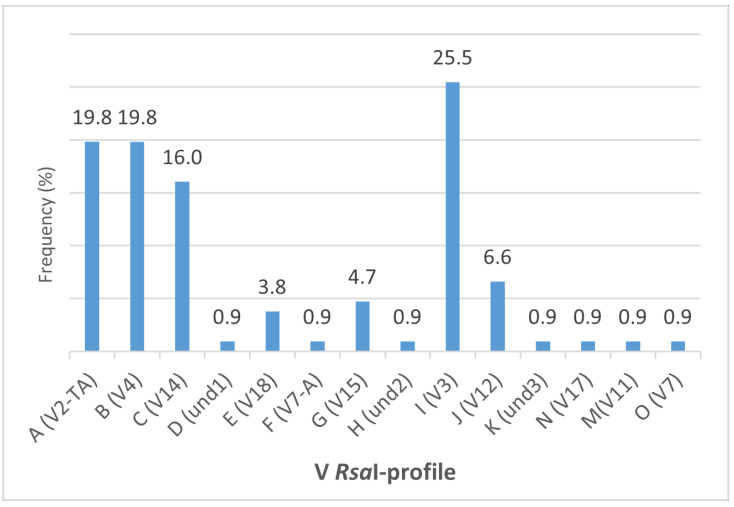
Distribution of the *Rsa*I-V *vmp1* profiles determined by restriction fragment length polymorphism (RFLP) analysis of *vmp1* amplicons in the 111 samples amplified.

The phylogenetic tree constructed using the *stamp* representative sequences showed the presence of two main *stamp* clusters, a and b, enclosing *tuf* type a (nettle-related) and *tuf* type b (bindweed-related) samples, respectively (Figure 3). The subcluster a-II enclosed *stamp* variants St8, St9, and St19 related to *tuf* type a sample (grapevine PM1, grapevine TB3, *Parthenocissus* 1). The subcluster a-I encompasses St11 *stamp* sequences enclosed in the *tuf* type b2 (grapevine 10). Moreover, grapevine TB11, *tuf* type b1, was enclosed within the subcluster a-I, while all the other *stamp* variants were enclosed in *stamp* b-I (St10), *stamp* b-II (St1, St2, St5), and *stamp* b-III (St3, St4) subclusters. The 25 lineages obtained by the combination of the restriction profiles of the tested genes were mainly discriminated by the *vmp1* gene that allowed differentiation of 15 variants (Table 2). V2-TA, V4, and V3 were the prevalent profiles, detected in 19.8%, 19.8%, and 25.5% of the samples, respectively (Figure 4). Furthermore, V2-TA, V3, V4, V12, and V14 profiles were detected in both grapevines and other species, whereas und1, und3, V11, and V18 were only detected in grapevines. Profiles und1 (Serbia), und2 (Slovenia), and und3 (Spain), detected only in grapevines, were unique, and differed from the already-described profiles. 

**Figure 5:**
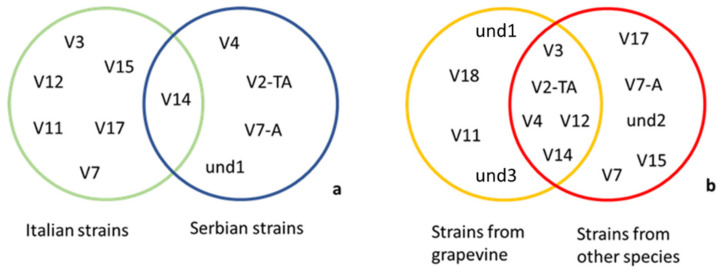
(**a**) *Vmp1*-sharing *Rsa*I-V profiles between Italian and Serbian strains; (**b**) *Vmp1*-sharing *Rsa*I-V profiles between ‘*Ca*. P. solani’ strains from grapevine and from other host species.

Moreover, one sample from tobacco from Serbia (strain 150/10) and one sample from grapevine from Italy (strain RA 9827) showed mixed profiles (Table 2). Most of the samples tested originated from Serbia and Italy, and the distribution of the different *vmp1* RFLP profiles showed that only the V14 profile was detected in both countries. Considering all the samples tested, five *vmp1* profiles could be detected both in grapevines and in other host species (profiles V2-TA, V3, V4, V12, and V14) (Figure 5). 

## 4. Discussion

The genetic variability of the ‘*Ca*. P. solani’ strains and the broad range of different plant host species infected are the key points in the study of population genetic and ecology of this phytoplasma. To provide an overall insight into its genetic variability and host distribution, two membrane protein coding genes (*vmp1* and *stamp*) involved in the recognition and interaction with its hosts [15,38] were studied. They showed a high sequence variability that make them useful to study the phytoplasma population dynamics. Moreover, the study of the elongation factor Tu (*tuf*) gene allowed the distinction of three variants (*tuf* type a, *tuf* type b2 and *tuf* type b1) involved in two BN disease cycles [19,20], while no other *tuf* variants were found [23,25,46]. Additionally, the first identification of a *tuf* type a (nettle-associated type) in naturally infected *P. quinquefolia* and *P. tricuspidata* added new host plant species to this ‘*Ca*. P. solani’ *tuf* type and indicated its possible involvement in alternative epidemiological cycles with different and previously undescribed, host species. 

The polymorphisms detected in the *stamp* gene improved the knowledge of the phytoplasma strain population structure and dynamics. Currently, 70 nucleotide sequence variants have been described [19,26,31,32,44,45], and the two new variants detected in this work, together with the 11 already published, confirmed the large genetic variability of this gene. The definition of the *stamp* variants showed that the genetic variability of this gene could be underestimated and not fully exploited by the RFLP analysis alone, since variants are often characterized by small inserts or deletions, not detected by the restriction analysis. Considering the *vmp1* gene, the host species distribution of V2-TA and V4 profiles was quite wide, since the first was detected in corn, grapevine, potato, tomato, parsley, and parsnip; while the latter was identified in bindweed, carrot, grapevine, potato, tomato, parsley, periwinkle, parsnip, tobacco, and valerian. The *vmp1* V4 profile was detected in grapevine samples from Italy, Croatia, Serbia, and Bosnia and Herzegovina [11,27,29,47,48,49]. On the contrary, the V3 profile showed a host species distribution limited to grapevine, *H. obsoletus*, *P. quinquefolia*, and *P. tricuspidata* from Italy, and it was detected in all the strains *tuf* type a, only in Italian samples. The presence of V14 profile in potato, grapevine, bindweed, celery, parsley, periwinkle, pepper, and valerian was confirmed mainly in Eastern European countries, confirming previous reports [17,31]. Out of the 17 samples in which it was identified (from Italy, Serbia, Montenegro, and Hungary), it was detected only in one grapevine sample from Central Italy (strain J1 from the Marche region). 

This study indicated that the variability and, in some cases, the unique combination of the environmental and agroecological conditions, play an important role in the strain selection, making them prevalent and/or endemic in a specific geographic area. The presence of previously unreported *vmp1* RFLP patterns (und1, und2, and und3) demonstrated the high degree of plasticity of this gene, which suggests further studies to fully understand its complexity and variability in this phytoplasma. However, studies focusing on correlation between different symptomatology and strain variability are still necessary to confirm the presence of virulent or mild strains in the diverse host species. 

While the high variability of the *vmp1* gene has proven to be useful for discriminating ‘*Ca*. P. solani’ lineages, the results of this study indicated that the epidemiology of this phytoplasma is more complex than already shown, since strains connected to nettle and grapevine cycle [26] have been identified in new host species. Despite that the strains analyzed in this work were collected in different years and countries, the variability detected showed incomplete consistency with the year or the country of collection. However, the lineage I was detected from 2005 to 2020 only in grapevines and *Parthenocissus* spp. and in Italian cultivations, while the lineage III was only identified in Serbia and Hungary from 2009 to 2018 in diverse plant host species. The lineage IV was identified in diverse host species only in Serbia until 2013, but in 2016 and 2017, it was also identified in Italy and Montenegro, and the lineage IX was only retrieved in 2009–2013 in a few host species in Serbia and Bulgaria. This survey’s results confirmed that the plasticity of these genes can be connected to both year and location of collection; however, comparable analyses of more ‘*Ca.* P. solani’ strains should be done to confirm the epidemiological trends indicated by the identified lineage diversity.

Asymptomatic, infected propagation material trade, due to the lack of screening and certification protocols, and the ability of diverse insect vectors to transmit ‘*Ca*. P. solani’ in the different geographical regions, are jeopardizing molecular-based epidemiological studies. It is nevertheless very important to continue the molecular monitoring of the ‘*Ca*. P. solani’ populations to verify the possible emergence or re-emergence and spread of epidemic strains of the pathogen also identified for their genetic homogeneity in the studied genes.

## Figures and Tables

**Table 1:** Sequences retrieved from the GenBank of *vmp1* gene variants used in the phylogenetic analysis.

Strain	Host	Country	GenBank Acc. No.
Mp46	Grapevine	Italy	HM008606
Char8	Grapevine	Georgia	KT184870
MK29	Grapevine	Macedonia	KF957604
STOL	Pepper	Serbia	AM992103
GGY	Grapevine	Germany	AM992102
Moliere	*Prunus avium*	France	AM992096
Rqg42	*R. quinquecostatus*	Serbia	KC703030
CrHo12_650	*H. obsoletus*	Austria	KJ469725
N9	Nettle	France	JQ977729
Aa25	Grapevine	Italy	HM008614
N3	Nettle	Slovenia	JQ977723
149/11	Grapevine	Italy	KJ145347
B7	Grapevine	Italy	HM008608
Mp49	Grapevine	Italy	HM008607
CrH12_721	*H. obsoletus*	Austria	KJ469731
36861_SLO_C	Bindweed	Slovenia	JQ977741
CH1	Grapevine	Italy	AM992105
PO	*H. obsoletus*	France	AM992095
Mvercer2	Grapevine	Italy	HM008612

**Table 2:** RFLP profiles and lineages obtained on the genes of *‘Ca.* P. solani’-infected samples analyzed. Identical background color means identical or possibly identical genotype.

Samples	Host	Location	Year	*Tuf*	*Stamp*	*Vmp1*	Lineage
*Parthenocissus* C	Virginia creeper	Italy	2005	A	E	I(V3)	I
Grapevine FC 10044	Grapevine	Italy	2010	A	E(St19)	I(V3)	I
Grapevine BO 9866	Grapevine	Italy	2010	A	E(St19)	I(V3)	I
Grapevine FE 9805	Grapevine	Italy	2010	A	E(St8)	I(V3)	I
Grapevine RA 9802	Grapevine	Italy	2010	A	E(St19)	I(V3)	I
*Parthenocissus* 1	Virginia creeper	Italy	2018	A	E(st19)	I(V3)	I
Grapevine PM1	Grapevine	Italy	2019	A	E(st8)	I(V3)	I
Grapevine TB1	Grapevine	Italy	2020	A	E	I(V3)	I
Grapevine TB10	Grapevine	Italy	2020	A	E	I(V3)	I
Grapevine TB2	Grapevine	Italy	2020	A	E	I(V3)	I
Grapevine TB4	Grapevine	Italy	2020	A	E	I(V3)	I
Grapevine TB7	Grapevine	Italy	2020	A	E	I(V3)	I
Grapevine TC4	Grapevine	Italy	2020	A	E	I(V3)	I
Grapevine TC6	Grapevine	Italy	2020	A	E	I(V3)	I
Grapevine TB3	Grapevine	Italy	2020	A	E(St9)	I(V3)	I
*P. tricuspidata* S	Boston Ivy	Italy	2020	A(mix)	E	I(V3)	I
Grapevine I6	Grapevine	Hungary	2008	B	A(St5)	A(V2-TA)	II
Tomato 127	Tomato	Hungary	2008	B	A	A(V2-TA)	II
Parsley 228/09	Parsley	Serbia	2009	B	A	A(V2-TA)	II
Parsley 231/09	Parsley	Serbia	2009	B	A	A(V2-TA)	II
Pepper 101/10	Pepper	Serbia	2010	B	A	A(V2-TA)	II
Parsnip 153/16	Parsnip	Serbia	2016	B	A	A(V2-TA)	II
MOL	Periwinkle	France	*	B	A	B(V4)	III
Potato N126a	Potato	Hungary	2008	B	A(St5)	B(V4)	III
Grapevine 190/09	Grapevine	Serbia	2009	B	A	B(V4)	III
Parsley 226/09	Parsley	Serbia	2009	B	A	B(V4)	III
Valeriana 262/09	Valerian	Serbia	2009	B	A	B(V4)	III
Carrot 89/10	Carrot	Serbia	2010	B	A(St1)	B(V4)	III
Grapevine 138/10	Grapevine	Serbia	2010	B	A	B(V4)	III
Periwinkle 202/10	Periwinkle	Serbia	2010	B	A(St1)	B(V4)	III
Periwinkle 80/10	Periwinkle	Serbia	2010	B	A(St1)	B(V4)	III
Tobacco 111/10	Tobacco	Serbia	2010	B	A	B(V4)	III
Tobacco 159/10	Tobacco	Serbia	2010	B	A	B(V4)	III
Bindweed 79/11	Bindweed	Serbia	2011	B	A	B(V4)	III
Grapevine 69/11	Grapevine	Serbia	2011	B	A(St1)	B(V4)	III
Periwinkle 97/11	Periwinkle	Serbia	2011	B	A(St1)	B(V4)	III
Parsnip 161/16	Parsnip	Serbia	2016	B	A	B(V4)	III
Bindweed 113/18	Bindweed	Serbia	2018	B	A(St2)	B(V4)	III
Potato N126b	Potato	Hungary	2008	B	A(St5)	C(V14)	IV
Periwinkle 147/09	Periwinkle	Serbia	2009	B	A(St2)	C(V14)	IV
Valeriana 224/09	Valerian	Serbia	2009	B	A	C(V14)	IV
Grapevine 122/10	Grapevine	Serbia	2010	B	A	C(V14)	IV
Grapevine 124/10	Grapevine	Serbia	2010	B	A	C(V14)	IV
Grapevine 134/10	Grapevine	Serbia	2010	B	A	C(V14)	IV
Parsnip 162/16	Parsnip	Serbia	2010	B	A	C(V14)	IV
Pepper 100/10	Pepper	Serbia	2010	B	A	C(V14)	IV
Bindweed 81/11	Bindweed	Serbia	2011	B	A	C(V14)	IV
Grapevine 66/11	Grapevine	Serbia	2011	B	A(St1)	C(V14)	IV
Grapevine 113/12	Grapevine	Serbia	2012	B	A	C(V14)	IV
Celery 252/13	Celery	Serbia	2013	B	A	C(V14)	IV
Celery 299/13	Celery	Serbia	2013	B	A(St2)	C(V14)	IV
Grapevine J1	Grapevine	Italy	2016	B	A(St1)	C(V14)	IV
Bindweed 284/17	Bindweed	Montenegro	2017	B	A(St1)	C(V14)	IV
Pepper 223/17	Pepper	Serbia	2017	B	A	F(V7-A)	V
Tomato Ca a	Tomato	Italy	2017	B	A(St5)	G(V15)	VI
Tomato Ca 1a	Tomato	Italy	2017	B	A	G(V15)	VI
Tomato Ca b	Tomato	Italy	2017	B	A(St5)	G(V15)	VI
Grapevine BO 9867	Grapevine	Italy	2010	B	A(St5)	J(V12)	VII
Grapevine TC7	Grapevine	Italy	2020	B	A	J(V12)	VII
Grapevine TC1	Grapevine	Italy	2020	B	A(St5)	J(V12)	VII
Tomato ORII	Tomato	Italy	2021	B	A(St5)	J(V12)	VII
*H. obsoletus* 72D	*H. obsoletus*	Italy	2019	B	A(St1)	N(V17)	VIII
Carrot 154/09	Carrot	Serbia	2009	B	B(St4)	A(V2-TA)	IX
Corn 121/09	Corn	Serbia	2009	B	B	A(V2-TA)	IX
Corn 107/09	Corn	Serbia	2009	B	B(St4)	A(V2-TA)	IX
Parsley 149/09	Parsley	Serbia	2009	B	B	A(V2-TA)	IX
Grapevine 123/10	Grapevine	Serbia	2010	B	B	A(V2-TA)	IX
Grapevine 120/12	Grapevine	Serbia	2012	B	B	A(V2-TA)	IX
Celery 251/13	Celery	Serbia	2013	B	B	A(V2-TA)	IX
Corn 241/13	Corn	Bulgaria	2013	B	B	A(V2-TA)	IX
Corn 244/13	Corn	Bulgaria	2013	B	B	A(V2-TA)	IX
Corn 263/13	Corn	Serbia	2013	B	B	A(V2-TA)	IX
Corn 242/13	Corn	Bulgaria	2013	B	B(St4)	A(V2-TA)	IX
Grapevine 189/09	Grapevine	Serbia	2009	B	B(St3)	C(V14)	X
Parsley 150/09	Parsley	Serbia	2009	B	B	C(V14)	X
Grapevine 144/10	Grapevine	Serbia	2010	B	B	D(^b^ und1)	XI
Grapevine 165/12	Grapevine	Bulgaria	2012	B	B	E(V18)	XII
Tomato P2	Tomato	Portugal	1998	B	B	G(V15)	XIII
Grapevine 3S	Grapevine	Spain	2018	B	B	K(^b^ und3)	XIV
Parsnip 152/16	Parsnip	Serbia	2016	B	D	A(V2-TA)	XIX
Grapevine TC5	Grapevine	Italy	2020	B	B(St10)	M(V11)	XV
STOL	Periwinkle	Serbia	*	B	B(St4)	A(V2-TA)	XVI
Tomato Herd	Tomato	Portugal	1998	B	C	B(V4)	XVII
Tomato P3	Tomato	Portugal	1998	B	C	B(V4)	XVII
Tomato Herd 4	Tomato	Portugal	1998	B	C	G(V15)	XVIII
Grapevine TB11	Grapevine	Italy	2020	B	D	J(V12)	XX
Grapevine CC2	Grapevine	Italy	2019	B	D(St18)	J(V12)	XX
Tomato P	Tomato	Portugal	1998	B	E	B(V4)	XXI
Grapevine I9	Grapevine	Hungary	2008	B	E	E(V18)	XXII
Grapevine I10	Grapevine	Hungary	2008	B	E(St11)	E(V18)	XXII
Grapevine I8	Grapevine	Hungary	2008	B	E(St11)	E(V18)	XXII
ASLO	Periwinkle	Slovenia	*	B	E	H(^b^und2)	XXIII
Grapevine FE 9806	Grapevine	Italy	2010	B	E	I(V3)	XXIV
P-TV	Periwinkle	Italy	*	B	A	O(V7)	XXV
STOF	Periwinkle	France	*	B	A	-	n.d.
Tomato P3	Tomato	Portugal	1997	B	B	-	n.d.
Tomato P4	Tomato	Portugal	1997	B	B(St10)	-	n.d.
Grapevine CHCA1	Grapevine	Italy	2015	-	E(St8)	-	n.d.
Grapevine J2	Grapevine	Italy	2016	-	A(St1)	-	n.d.
Grapevine C1	Grapevine	Italy	2016	B	A(St1)	-	n.d.
Grapevine GY5	Grapevine	Italy	2018	B	A(St1)	-	n.d.
Grapevine TC3	Grapevine	Italy	2020	B	-	-	n.d.
Potato N128a	Potato	Hungary	2008	B	-	A(V2-TA)	n.d.
Potato N128b	Potato	Hungary	2008	B	-	A(V2-TA)	n.d.
Grapevine RA 9827	Grapevine	Italy	2010	B	A	^a^ mix	n.d.
Tobacco 150/10	Tobacco	Serbia	2010	B	B(St3)	^a^ mix	n.d.
Tomato N130	Tomato	Hungary	2008	B	-	B(V4)	n.d.
Valeriana 222/09	Valerian	Serbia	2009	-	A	B(V4)	n.d.
Grapevine RA 9709	Grapevine	Italy	2010	A	-	I(V3)	n.d.
Grapevine CHSM2	Grapevine	Italy	2015	-	E(St19)	I(V3)	n.d.
Grapevine CS2	Grapevine	Italy	2019	-	-	I(V3)	n.d.
Grapevine GM3	Grapevine	Italy	2019	-	-	I(V3)	n.d.
*H. obsoletus* 67C	*H. obsoletus*	Italy	2019	-	E	I(V3)	n.d.
*H. obsoletus* 67F	*H. obsoletus*	Italy	2019	-	E(St19)	I(V3)	n.d.
Grapevine PM2	Grapevine	Italy	2019	-	E(St8)	I(V3)	n.d.
Grapevine TB12	Grapevine	Italy	2020	-	E	I(V3)	n.d.
Grapevine TB5	Grapevine	Italy	2020	-	E	I(V3)	n.d.
Grapevine TC2	Grapevine	Italy	2020	A	-	I(V3)	n.d.
Grapevine TB8	Grapevine	Italy	2020	-	B	J(V12)	n.d.

Note: -, negative to PCR amplification; n.d., not done: lineage not determined for lack of all genes amplification; ^a^ mix, mixed *Rsa*I-RFLP profile; ^b^ und, undescribed *Rsa*I restriction profile; *, strain from collection.

**Table 3:** Samples sequenced and used for the phylogenetic analyses.

Samples	Origin	*Tuf* Variant	GenBank Acc. No	*Stamp* Profile	*Stamp* Variant	GenBank Acc. No.	*Vmp1* Profile	GenBank Acc. No.
Grapevine 138/10	Serbia	b1	/	A	/	/	B(V4)	+
Pepper 223/17	Serbia	b1	/	A	/	/	F(V7-A)	MW791438
P-TV	Italy	b1	/	A	/	/	O(V7)	MW791437
Grapevine 3S	Spain	b1	MZ970611	B	/	MW759856	K(und3)	MW791432
Celery 251/13	Serbia	b1	/	B	/	/	A(V2-TA)	+
Tomato Herd 4	Portugal	b1	/	C	/	+	G(V15)	MW791434
Tomato P3	Portugal	b1	/	C	/	MW759855	B(V4)	/
Grapevine J2	Italy	-	-	A	St1	+	-	-
Bindweed 284/17	Montenegro	b1	/	A	St1	+	C(V14)	+
Carrot 89/10	Serbia	b1	/	A	St1	+	B(V4)	+
*H. obsoletus* 72D	Italy	b1	/	A	St1	MW759854	N(V17)	MW791433
Periwinkle 202/10	Serbia	b1	/	A	St1	+	B(V4)	+
Periwinkle 80/10	Serbia	b1	/	A	St1	+	B(V4)	MW791442
Grapevine C1	Italy	b1	/	A	St1	+	-	-
Grapevine GY5	Italy	b1	MZ970610	A	St1	+	-	-
Grapevine J1	Italy	b1	/	A	St1	+	C(V14)	/
Grapevine 69/11	Serbia	b1	/	A	St1	+	B(V4)	/
Grapevine 66/11	Serbia	b1	/	A	St1	+	C(V14)	/
Grapevine TC5	Italy	b1	/	B	St10	MW759860	M(V11)	/
Tomato P4	Portugal	b1	/	B	St10	+	-	-
Grapevine I10	Hungary	b2	MZ970609	E	St11	MW759861	E(V18)	MW791439
Grapevine I8	Hungary	b2	MZ970607	E	St11	+	E(V18)	+
Grapevine CC2	Italy	b1	MZ970606	D	St18	+	J(V12)	+
Grapevine TB11	Italy	b1	MW755980	D	St18	MW759859	J(V12)	+
*H. obsoletus* 67F	Italy	-	-	E	St19	+	I(V3)	+
Grapevine CHSM2	Italy	-	-	E	St19	+	I(V3)	+
Grapevine RA 9802	Italy	a	/	E	St19	+	I(V3)	+
Grapevine BO 9866	Italy	a	/	E	St19	+	I(V3)	+
Grapevine FC 10044	Italy	a	/	E	St19	+	I(V3)	+
*Parthenocissus* 1	Italy	a	MZ970608	E	St19	OL412284	I(V3)	+
Bindweed 113/18	Serbia	b1	/	A	St2	OL412285	B(V4)	+
Celery 299/13	Serbia	b1	/	A	St2	+	C(V14)	+
Periwinkle 147/09	Serbia	b1	/	A	St2	+	C(V14)	MW791440
Grapevine 189/09	Serbia	b1	/	B	St3	MW759852	C(V14)	/
Tobacco 150/10	Serbia	b1	/	B	St3	+	mix	/
Carrot 154/09	Serbia	b1	/	B	St4	+	A(V2-TA)	+
Corn 242/13	Bulgaria	b1	/	B	St4	MW759851	A(V2-TA)	MW791441
Corn 107/09	Serbia	b1	/	B	St4	+	A(V2-TA)	/
Grapevine BO 9867	Italy	b1	/	A	St5	MW759857	J(V12)	MW791436
Tomato ORII	Italy	b1	+	A	St5	+	J(V12)	+
Grapevine I6	Hungary	b1	/	A	St5	+	A(V2-TA)	/
Grapevine TC1	Italy	b1	/	A	St5	+	J(V12)	/
Potato N126a	Hungary	b1	/	A	St5	+	B(V4)	/
Potato N126b	Hungary	b1	/	A	St5	+	C(V14)	/
Tomato Ca a	Italy	b1	/	A	St5	+	G(V15)	/
Tomato Ca b	Italy	b1	/	A	St5	+	G(V15)	/
Grapevine PM2	Italy	-	-	E	St8	+	I(V3)	+
Grapevine CHCA1	Italy	-	-	E	St8	+	-	-
Grapevine PM1	Italy	a	/	E	St8	MW759853	I(V3)	+
Grapevine FE 9805	Italy	a	/	E	St8	+	I(V3)	+
Grapevine TB3	Italy	a	MW755979	E	St9	MW759858	I(V3)	MW791435

Note: -, negative samples; /, samples not sequenced, and variants not determined; +, samples sequenced.

## Data Availability

The archived datasets analyzed or generated during this study were deposited in the NCBI GenBank.
